# Neurogenic differentiation factor NeuroD confers protection against radiation-induced intestinal injury in mice

**DOI:** 10.1038/srep30180

**Published:** 2016-07-20

**Authors:** Ming Li, Aonan Du, Jing Xu, Yanchao Ma, Han Cao, Chao Yang, Xiao-Dong Yang, Chun-Gen Xing, Ming Chen, Wei Zhu, Shuyu Zhang, Jianping Cao

**Affiliations:** 1 School of Radiation Medicine and Protection, Medical College of Soochow University, Suzhou 215123, China; 2 Collaborative Innovation Center of Radiation Medicine of Jiangsu Higher Education Institutions, Soochow University, Suzhou 215123, China; 3 Department of Transfusion Medicine, The General Hospital of the PLA Rocket Force, Beijing 100088, China; 4 Department of General Surgery, The Second Affiliated Hospital of Soochow University, Suzhou 215004, China; 5 Zhejiang Key Laboratory of Radiation Oncology, Zhejiang Cancer Hospital, Hangzhou 310022, China

## Abstract

The gastrointestinal tract, especially the small intestine, is particularly sensitive to radiation, and is prone to radiation-induced injury as a result. Neurogenic differentiation factor (NeuroD) is an evolutionarily-conserved basic helix-loop-helix (bHLH) transcription factor. NeuroD contains a protein transduction domain (PTD), which allows it to be exogenously delivered across the membrane of mammalian cells, whereupon its transcription activity can be unleashed. Whether NeuroD has therapeutic effects for radiation-induced injury remains unclear. In the present study, we prepared a NeuroD-EGFP recombinant protein, and explored its protective effects on the survival and intestinal damage induced by ionizing radiation. Our results showed that NeuroD-EGFP could be transduced into small intestine epithelial cells and tissues. NeuroD-EGFP administration significantly increased overall survival of mice exposed to lethal total body irradiation (TBI). This recombinant NeuroD also reduced radiation-induced intestinal mucosal injury and apoptosis, and improved crypt survival. Expression profiling of NeuroD-EGFP-treated mice revealed upregulation of tissue inhibitor of metalloproteinase 1 (TIMP-1), a known inhibitor of apoptosis in mammalian cells. In conclusion, NeuroD confers protection against radiation-induced intestinal injury, and provides a novel therapeutic clinical option for the prevention of intestinal side effects of radiotherapy and the treatment of victims of incidental exposure.

The application of radiation and radioactive compounds in agricultural and medical technologies have afforded enormous benefits to humankind, but overexposure to the ionizing radiation can cause acute radiation syndrome (ARS), posing a complex medical challenge^1^. Furthermore, ARS may arise from the fallout of nuclear accidents and terrorism, necessitating an improvement in our understanding and treatment^2^. Whilst the molecular etiology underlying ARS remains complex and largely unknown, a few radioprotective drugs have proved successful in clinical practice. Amifostine (WR-2721; (2-(3-aminopropylamino) ethylsulphanyl phosphonic acid) is an organic thiophosphate cytoprotective agent, and was the first radioprotective drug applied in clinical practice. Furthermore, a combination of pentoxifylline and tocopherol has demonstrated potential as radioprotectors or radiomitigators^3^. Nevertheless, these radioprotective drugs may induce severe side effects in patients, limiting their application. Therefore, exploring the molecular events of ARS, and developing effective therapeutic treatments, is urgently needed to improve the outcomes of radiation-induced injuries.

The gastrointestinal tract, especially the small intestine, is particularly sensitive to radiation, rendering it vulnerable to the effects of collateral radiation from the radiotherapeutic treatment of abdominal and pelvic cancers^4^,^5^. Histologically, overexposure to ionizing radiation (IR) may result in the shortening of villi, disruption to the mucosal architecture, or even apoptosis and necrosis of the intestinal crypts^6^. The effects may manifest clinically as vomiting, diarrhea, malabsorption and radiation enteritis^7^. Currently, there are no effective clinical treatments for radiation-induced intestinal injury.

Transcription factors are essential to multiple physiological and pathological processes, serving as molecular switches that turn specific sets of genes on or off^8^. The neurogenic differentiation factor (NeuroD), also known as β-cell E-box Trans-activator 2 (BETA2), is an evolutionarily-conserved basic helix-loop-helix (bHLH) transcription factor^9^. The human NeuroD gene is located in the chromosome 2q32, and is highly expressed in pancreatic, intestinal and brain tissues^10^. Murine NeuroD is 88.5% identical to the human counterpart. NeuroD has been demonstrated to regulate multiple genes involved in cell cycle progression, cell fate determination and cellular differentiation^11^,^12^,^13^. Consequently, the knockout of NeuroD in mice defects pancreatic morphogenesis^14^ and causes neural defects in the granule layers of the cerebellum and hippocampus^15^, indicating that this gene is critical in individual development. The expression of NeuroD is also required during the earliest stages of islet formation development and for the secretion of insulin in mature β-cells upon glucose stimulation^11^,^12^,^13^. Additionally, NeuroD contains its own protein transduction domain (PTD), enabling it to cross the membrane of mammalian cells^16^,^17^. The arginine- and lysine-rich 14 Aa peptide ‘KPKRRGPKKKKMTK’ and the C-terminal amphipathic helix in the bHLH domain are essential for the protein transduction capability of NeuroD. The internalized NeuroD protein still preserves its transcription activity^16^,^17^.

Our previous research demonstrated that supplementation of exogenous NeuroD protein can be transduced into the small intestine epithelium cells post intraperitoneal injection, thereby alleviating the symptoms of diabetes mellitus within a mouse model induced by enteric expression of insulin^18^. Thus, the possibility that intestinal NeuroD is modulated by radiation, and the use of NeuroD for the therapeutic treatment of radiation-induced intestinal injury, remain unclear. In this study, we synthesized a NeuroD-EGFP recombinant protein and explored its role in radiation-induced damage. Our results showed that NeuroD-EGFP administration significantly increased the overall survival of mice exposed to lethal total-body irradiation (TBI) and ameliorated radiation-induced intestinal mucosal injury by upregulating anti-apoptotic tissue inhibitor of metalloproteinase 1 (TIMP-1).

## Results

### Induction of NeuroD expression following different doses of radiation

**Figure 1:**
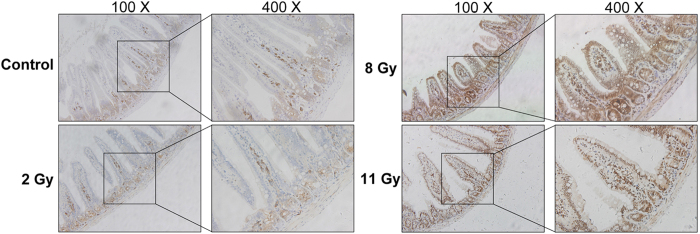
The levels and cellular localization of NeuroD in small intestinal epithelia of C57BL/6J mice after varied doses of TBI. Immunohistochemical staining of NeuroD in paraffin sections from small intestines of control (0 Gy) mice or those after different doses of TBI (100x and 400x magnification). TBI of 2, 8 or 11 Gy was delivered to C57BL/6J mice using a 6-MeV electron linear accelerator at a fixed dose rate of 2 Gy per minute.

To investigate whether NeuroD expression is altered in response to radiation, C57BL/6J mice were exposed to different doses of TBI, whereupon the small intestines were collected and the expression of NeuroD was determined 6 h after irradiation. The results revealed that NeuroD was expressed in the central lacteal villi of the small intestine. Treatment with 8 and 11 Gy irradiation markedly increased the expression of NeuroD protein in the intestine of mice relative to the sham irradiated control mice (Fig. 1). The increased NeuroD was distributed in the crypts and epithelia of mice small intestines. These results suggested that NeuroD may be involved in the response of the murine small intestine to irradiation.

### Preparation of NeuroD-EGFP and its transduction into small intestine epithelium cells and tissues

**Figure 2:**
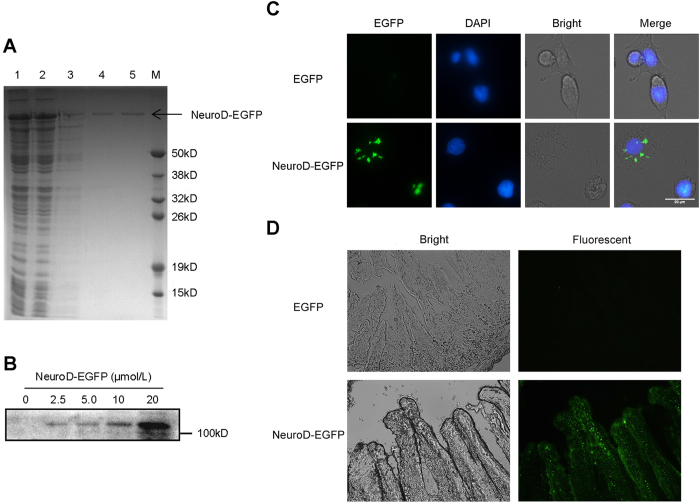
Preparation of NeuroD-EGFP and its transduction into small intestinal epithelium cells *in vitro* and *in vivo*. (**A**) Purified NeuroD-EGFP fusion proteins were analyzed by 12% SDS-PAGE and stained with Coomassie Blue. Lanes are as follows: lane 1, supernatant after sonication; lane 2, effluent after loading; lane 3, 100 mM imidazole elution buffer; lane 4, 200 mM imidazole elution buffer; lane 5, 300 mM imidazole elution buffer; lane M, protein marker. **(B)** Purified NeuroD-EGFP fusion proteins were analyzed by Western blot. **(C)** Transduction of NeuroD-EGFP into IEC-6 rat jejunal crypt cell line. Nuclear DNA (blue) was stained with DAPI. Scale bar: 20 μm. **(D)** Transduction of NeuroD-EGFP into mouse small intestine (400x magnification).

To explore the potential role of NeuroD in radiation response, purified NeuroD protein was fused with EGFP at the C-terminal to obtain a NeuroD-EGFP fusion protein. As presented in Fig. 2A, this fusion protein was expressed at a very high level and was present in the supernatant of the whole cell lysate. The fusion protein was purified by affinity chromatography, and subsequently confirmed by Western blot (Fig. 2B).

Next, we examined the impact of NeuroD-EGFP transduction into the IEC-6 rat jejunal crypt cell line. The fluorescence activity of EGFP was used to track the location of the NeuroD protein. After seeding, culture medium of IEC-6 cells was added to either EGFP or NeuroD-EGFP. Five hours post incubation, the IEC-6 cells treated with natural EGFP showed a weak EGFP signal, whereas IEC-6 cells treated with NeuroD-EGFP showed increase in the intracellular localization of EGFP, as determined by fluorescence microscopy (Fig. 2C), indicating that NeuroD-EGFP fusion protein can be transduced into IEC-6 cells.

We further tested whether the NeuroD-EGFP fusion protein can preserve its transduction activity *in vivo*. Eight-week old C57BL/6J mice were injected intraperitoneally with NeuroD-EGFP protein (0.5 μmol/kg) or an equivalent dose of EGFP protein for negative controls. Five hours later, the C57BL/6J mice were sacrificed, and the 5-μm frozen sections of the small intestine were prepared and then analyzed for the intensity of EGFP. The small intestine of the EGFP-treated mice showed weak EGFP signal, whereas marked fluorescence was found in the small intestine of NeuroD-EGFP treated mice (Fig. 2D). These results indicate that NeuroD-EGFP can be transduced into the small intestine epithelium cells *in vivo*.

### NeuroD-EGFP improves survival of mice after lethal doses of irradiation

**Figure 3:**
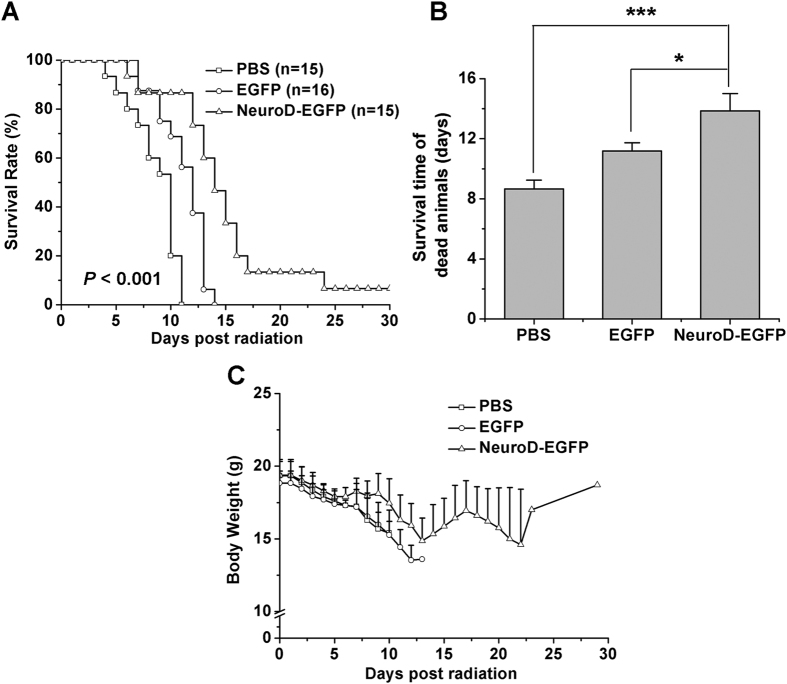
NeuroD-EGFP improves survival of mice after lethal doses of irradiation. (**A**) Kaplan-Meier survival analyses of C57BL/6J mice after 8 Gy TBI with or without NeuroD-EGFP treatment. **(B)** Mean survival time of dead animals in each group. *P < 0.05; ***P < 0.001. **(C)** Body weight change of C57BL/6J mice after 8 Gy TBI with or without NeuroD-EGFP treatment.

To investigate the effect of NeuroD-EGFP protein on the survival of irradiated mice, NeuroD-EGFP was immediately administered intraperitoneally into C57BL/6J mice treated with varying doses of TBI. Animal survival was monitored for up to 30 days. TBI at 8 Gy proved fatal in 100% of C57BL/6J mice treated with PBS or EGFP within 15 days, whereas treatment with NeuroD-EGFP significantly improved the survival rate and prolonged survival time of C57BL/6J mice (*P* < 0.05; Fig. 3A,B). However, the protective role of NeuroD-EGFP protein had on survival failed when the radiation dose was increased to 9 Gy (see Supplementary Fig. S1). It is known that exposure to TBI induced signs of radiation sickness, including diarrhea, black stools and weight loss^19^,^20^. NeuroD-EGFP administration alleviated radiation-induced weight loss in mice (Fig. 3C).

### NeuroD-EGFP mitigates intestine structural injury induced by TBI

**Figure 4:**
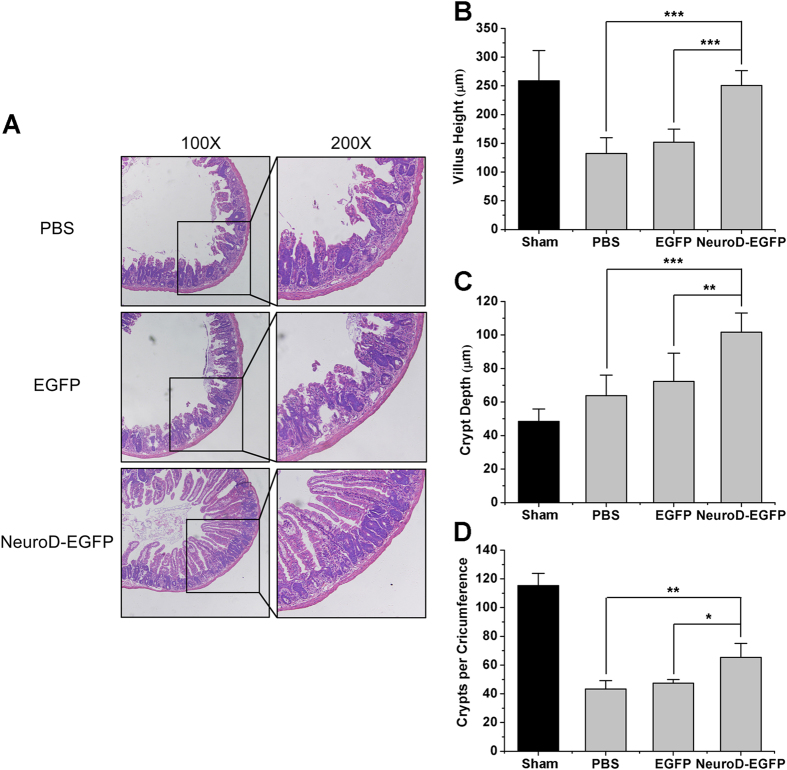
NeuroD-EGFP administration mitigates intestinal structural injury induced by 9 Gy TBI. **(A)** Representative H&E staining of intestinal sections from PBS, EGFP or NeuroD-EGFP treated mice at 3.5 days after 9 Gy TBI (100x or 200x magnification). Bar graph of villus height **(B)**, crypt depth **(C)**, and crypts per circumference **(D)** determined by measuring vertically well-oriented crypt villus units from H&E-stained sections of mice after 9 Gy TBI. Normal, sham-irradiated control. For villus height or crypt depth measurement, at least 30 villi or crypts per mouse were measured. Crypts per circumference were counted in 3 separate tubular intestinal slices for each mouse. *P < 0.05; **P < 0.01; ***P < 0.001; n = 3.

To examine the putative role NeuroD-EGFP protein has in protecting against radiation induced intestinal injury, we compared the histological manifestations within the small intestines of NeuroD-EGFP treated or untreated mice post 9 Gy TBI. At day 3.5, evidence of mucosal architecture destruction, such as villous denudation and crypt atrophy, was observed in the small intestine of PBS- or EGFP-treated mice, whereas the crypt-villi structures of the small intestine were preserved in NeuroD-EGFP-treated mice (Fig. 4A). Crypt-villi architecture restored in each group on day 7 post TBI (see Supplementary Fig. S2).

Villus heights, crypt depths and crypts per circumference at 3.5 days post TBI were also measured. Villus heights of PBS- or EGFP-treated mice reduced significantly following TBI compared to those of the sham-irradiated mice. Treatment with NeuroD-EGFP dramatically attenuated the radiation-induced reduction in villus heights (PBS: 132.08 ± 27.75 μm; EGFP: 151.98 ± 22.65 μm; NeuroD-EGFP: 250.68 ± 25.94 μm; *P* < 0.05; Fig. 4B). Crypt depths in the NeuroD-EGFP-treatment group (101.72 ± 11.43 μm) were greater than that in PBS (63.85 ± 12.28 μm) or EGFP treated mice (72.29 ± 16.91 μm; *P* < 0.05; Fig. 4C). There was a dramatic decrease in crypts per circumference in mice treated with PBS or EGFP compared with sham-irradiated mice, whereas NeuroD-EGFP-treated mice showed more crypts per circumference than that in the PBS or EGFP treatment groups (*P* < 0.05; Fig. 4D). Taken together, these findings indicate that NeuroD-EGFP administration protects against radiation-induced intestine structural injuries. Though, NeuroD-EGFP administration had no effect on the survival of C57BL/6J mice receiving 9 Gy TBI.

### NeuroD-EGFP promotes cell proliferation and reduces apoptosis in intestinal crypts after 9 Gy TBI

**Figure 5:**
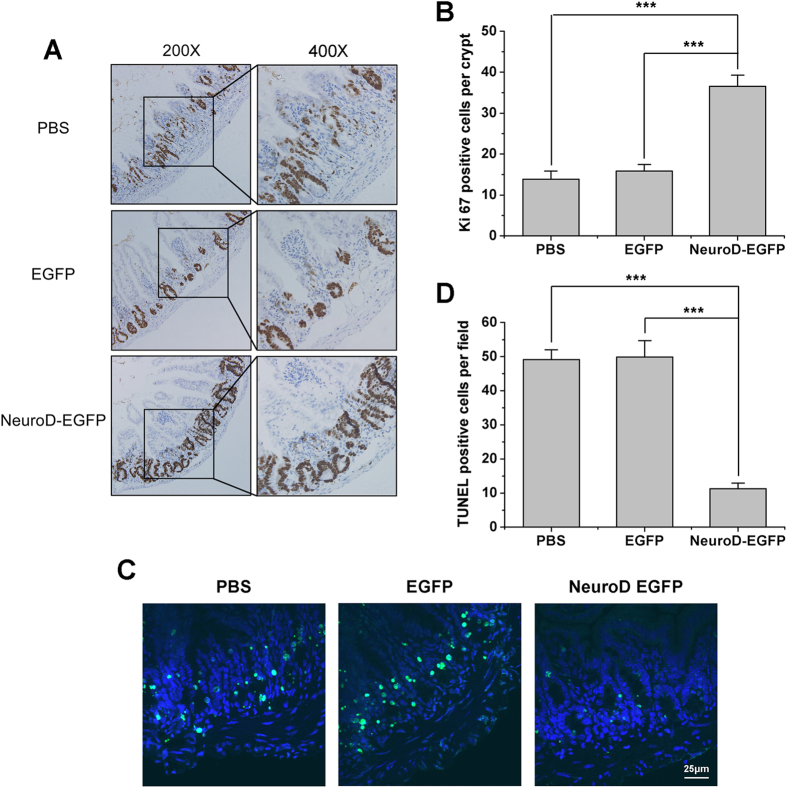
NeuroD-EGFP augments cell proliferation and reduces cell apoptosis in intestinal crypt epithelia after 9 Gy TBI. **(A)** Representative Ki67-immunostained intestinal sections from PBS, EGFP or NeuroD-EGFP treated mice at 3.5 days after 9 Gy TBI (200x or 400x magnification). **(B)** Quantification of Ki67-positive cells per crypt determined from panel (A). At least 30 well-oriented crypts per mouse were counted. ***P < 0.001; n = 3. **(C)** Representative TUNEL-staining of intestinal sections from PBS, EGFP or NeuroD-EGFP treated mice at 6 hours after 9 Gy TBI. TUNEL-stained epithelium shows green fluorescence. Nuclear DNA (blue) was stained with DAPI. Scale bar: 25 μm. **(D)** Quantification of TUNEL-positive cells determined from panel (C). The average number of positive cells in ten fields (400x magnification) per treated mouse was determined. *P < 0.05; n = 3.

The small intestinal sections were examined by immunohistochemistry using an antibody against Ki67 to assess the proliferation of crypt epithelial cells at 3.5 days post TBI. We observed an average of 13.83 and 15.83 Ki67-positive cells per crypt in PBS- and EGFP-treated mice, respectively. In the NeuroD-EGFP treated mice, an average of 36.55 Ki67-positive cells per crypt were observed (*P* < 0.001; Fig. 5A,B). Furthermore, TUNEL assay was performed to examine cell death of crypt epithelial cells in the small intestine at 6 h post TBI. As presented in Fig. 5C,D, the number of TUNEL-positive cells per field in NeuroD-EGFP treated mice was significantly lower than that in PBS- and EGFP-treated mice (*P* < 0.001). Thus, NeuroD-EGFP administration could promote crypt cell proliferation and reduce radiation-induced crypt cell death.

### NeuroD-EGFP ameliorates radiation-induced intestinal injury by upregulating anti-apoptotic TIMP-1

**Figure 6:**
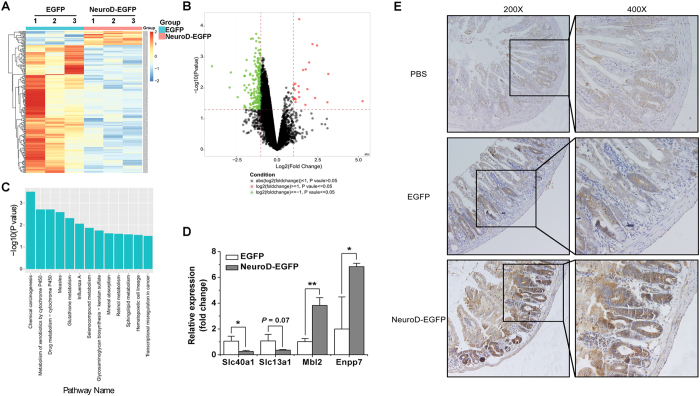
Gene expression profiling of jejunum samples of EGFP and NeuroD-EGFP treated mice. **(A)** Map of gene clusters for those differentially expressed between EGFP and NeuroD-EGFP treated mice at 12 hours post 9 Gy TBI. **(B)** Volcano plot comparing EGFP treatment versus NeuroD-EGFP treatment. Genes with fold change ≥2 and *P* value ≤ 0.05 are marked with red dots, while those with fold change ≤−2 and *P* value ≤ 0.05 are marked with green dots. **(C)** Significant pathways altered in mice 12 hours post 9 Gy TBI (EGFP treated mice *vs*. NeuroD-EGFP treated mice). **(D)** Quantitative real-time PCR validation for differentially expressed genes between EGFP and NeuroD-EGFP treated mice. *P < 0.05; **P < 0.01; n = 3. (**E**) NeuroD-EGFP induced TIMP-1 expression. The expression of TIMP-1 in the mice small intestine was determined by immunohistochemical staining with a TIMP-1 antibody (200x or 400x magnification).

To understand the underlying mechanisms responsible for NeuroD-EGFP-mediated protection from radiation-induced injury in murine radiosensitive intestinal tissues, we profiled gene that were differentially expressed in the small intestine in response to systemic administration of NeuroD-EGFP. C57BL/6J mice were treated intraperitoneally with NeuroD-EGFP or EGFP immediately after 9 Gy TBI. Twelve hours after the treatments, jejunum tissues were collected and microarrayed for more than 45,200-transcript assay probes. The results revealed a total of 231 differentially-expressed genes (*P* < 0.05) between EGFP- and NeuroD-EGFP treated mice. Among them, 21 genes were upregulated and 210 genes were downregulated (Fig. 6A–C and Table 1). The expression patterns of four representative genes (two upregulated genes Enpp7 and Mbl2, and two downregulated genes Slc13a1 and Slc40a1) were determined by qRT-PCR. The expression of Slc40a1, Enpp7 and Mbl2 in the four tested genes was consistent with the microarray assay (Fig. 6D).

Among these differentially expressed genes, Timp-1 (over 5-fold upregulation), a known inhibitor of apoptosis in mammalian cells, was studied further. We confirmed the upregulation of TIMP-1 in response to NeuroD-EGFP administration by immunohistochemistry analysis (Fig. 6E). Taken together, these results suggest that NeuroD-EGFP administration protects gut mucosal tissue from irradiation-induced apoptosis via TIMP-1.

## Discussion

The availability of agents to prevent or mitigate radiation-induced injuries is crucial for improving radiation therapy and our response to nuclear accidents^1^,^2^. At present, amifostine is the only cytoprotective agent approved by FDA for the prevention of radiation toxicity in humans^20^. The drug is usually administered before radiation, and causes many side effects, including hypocalcemia, hypotension, diarrhea, nausea, vomiting, immune hypersensitivity syndrome, erythroderma and anaphylaxis, which limit its application in the clinic^21^,^22^. Herein, we provide evidence that NeuroD is protective against radiation-induced intestinal injury when administered after radiation exposure, thereby providing a putative therapeutic option for the treatment of victims of accidental irradiation.

The small intestine is particularly radiosensitive, and yet there are no effective prophylactic or therapeutic treatments for IR-induced intestinal injury. In the present study, we found that the expression of NeuroD was induced in the small intestine of mice after radiation. We therefore purified a NeuroD-EGFP fusion protein from a bacterial system. The recombinant NeuroD-EGFP protein could be rapidly, and efficiently, transduced into the small intestinal epithelium cells *in vitro* and *in vivo*. Further experiments indicated that the fusion protein significantly enhanced survival of mice exposed to lethal TBI, reduced radiation-induced villous denudation and crypt atrophy, and protected intestinal mucosal architecture. Although the biological role of the PTD in endogenous NeuroD is still unknown, the bHLH PTD can mediate the internalization of exogenous NeuroD into target cells. Unlike vector- or virus-mediated gene editing, the application of recombinant proteins such as NeuroD can be well controlled in terms of application time and dosage, therefore avoiding unexpected adverse effects.

To understand the molecular mechanism of NeuroD-EGFP mediated radioprotection against radiation-intestinal injury, microarray analysis was performed, whereupon 21 upregulated and 210 downregulated genes were identified. As expected, NeuroD seems to exert its protective role via complex mechanisms affecting multiple genes and pathways, including TIMP-1. TIMP-1 has been reported to inhibit most of the known matrix metalloproteinases (MMPs), a group of proteases involved in the degradation of the extracellular matrix^22^. Moreover, TIMP-1 is able to promote cell proliferation in a wide range of cell types, including fibroblasts, keratinocytes, chondrocytes, epithelial cells, breast carcinoma cells and various leukemic cell lines^23^,^24^,^25^. Mechanistically, TIMP-1 promotes cell proliferation by activating the Ras/Raf/MAPK^26^,^27^ and PI3K/Akt pathways^28^. Beyond these activities, TIMP-1 has shown an anti-apoptotic function in different types of cells, including lymphoma cells, pancreatic islets, granulocytes and endothelial cells^29^,^30^,^31^,^32^. Multiple signaling pathways, including FAK/PI3K, ERK, JNK and NF-κB, were involved in the suppression of apoptosis by TIMP-1^30^,^33^,^34^,^35^. The pro-proliferative and anti-apoptotic activities of TIMP-1 may be at least partially responsible for the NeuroD-mediated reduction of cell apoptosis and augmentation of cell proliferation in intestinal crypts after 9 Gy TBI. Then, the question is how NeuroD regulates TIMP-1. NeuroD as a transcription factor that mediates transcriptional activation by binding to the E box of promoters with 5′-CANNTG-3′ consensus sequences^6^,^36^,^37^. However, this NeuroD binding motif was not found in the proximal promoter of TIMP-1, indicating that NeuroD may activate TIMP-1 indirectly. Consequently, the mechanism(s) enabling NeuroD to regulate TIMP-1 needs further investigation.

Stem cells of adult tissues are responsible for maintaining tissue homeostasis and regeneration following genotoxic stress or injuries^38^. Active intestinal stem cells contribute robustly to homeostatic regeneration, and are quantitatively ablated by irradiation. NeuroD has been reported to regulate the terminal differentiation of stem cells in various tissues. NeuroD, for example, was originally identified as a neurogenic differentiation factor in Xenopus^7^. Furthermore, NeuroD is indispensable for the differentiation of pancreatic stem cells^39^, induces differentiation of bone marrow mesenchymal stem cells into insulin-producing cells under given conditions^40^, and its upregulation via the bHLH transcription factor neurogenin 3 (Neurog3) appears to initiate endocrine differentiation in the gastrointestinal tract^41^. In this study, the protective role of NeuroD may be attributed partially to its stimulating role in the proliferation and partial differentiation of intestinal stem cells. However, we found that the PTD from NeuroD did not show specificity to certain types of cells. Innovation of an intestinal stem cell-specific delivery of the radioprotective NeuroD will be meaningful for the treatment of radiation-induced intestinal injury.

In conclusion, NeuroD confers protection against radiation-induced intestinal injury, and provides a therapeutic clinical option for the prevention of intestinal side effects of radiotherapy and the treatment of victims of incidental radiation exposure.

## Materials and Methods

### Construction of the NeuroD-EGFP expression vector

The NeuroD-EGFP (enhanced green fluorescent protein) expression vector was constructed according to the method described previously^18^. Briefly, the full-length NeuroD cDNA was amplified from mouse tissue by PCR. The PCR products were digested with BamHI and SalI, and inserted into BamHI and NotI sites of a pET-32a vector (Novagen, Madison, USA), along with the EGFP fragments, to generate a pET-32a-NeuroD-EGFP plasmid. The plasmid was confirmed by sequencing.

### Expression and purification of fusion protein

The constructed pET-32a-NeuroD-EGFP plasmid was transformed into an *Escherichia coli* BL-21 (DE3) strain, and then the successful transformants were selected on a LB plate containing ampicillin. When the OD_600_ (optical density at 600 nm) of BL21 (DE3) cells containing the expression plasmid reached 0.8 following incubation at 37 °C, 1.0 mM isopropyl-beta-D-thiogalactoside (IPTG) was added, and the cells were then incubated for an additional 12 h at 24 °C. Cells were sonicated, and the supernatants were recovered and applied to a Ni-nitrilotriacetic acid agarose column (Qiagen, Valencia, USA). After the NeuroD-EGFP had been absorbed by the Ni^2+^ column, the column was washed twice with PBS, and then the fusion protein was eluted with a gradient of imidazole elution buffer.

### Cell culture and treatment

Rat jejunal crypt cell line IEC-6 was purchased from the Cell Bank of the Chinese Academy of Sciences (Shanghai, China), and maintained in Dulbecco’s modified Eagle’s medium (DMEM) supplemented with 10% (v/v) heat-inactivated fetal bovine serum (FBS) and 1% (v/v) penicillin-streptomycin (Hyclone, Logan, USA) at 37 °C in a humidified atmosphere containing 5% CO_2_. IEC-6 cells were seeded onto a 96-well plate at a density of 1 × 10^4^ cells/well. After a 24-h incubation, the medium was replaced by fresh medium containing 1 μM of purified recombinant EGFP (Sangon Biotech, Shanghai, China) or NeuroD-EGFP protein. After five hours, the cells were washed twice by PBS. The cells were counterstained with 4,6-diamidino-2-phenylindole (DAPI; Sigma-Aldrich, St. Louis, USA) and imaged under a confocal microscope.

### Experimental animals and irradiation

Male C57BL/6 mice (8 weeks old, weighing 23~26 g) were purchased from Shanghai SLAC Laboratory Animal Co., Ltd. (Shanghai, China). Animals were housed under controlled conditions at a temperature of 20–22 °C, a relative humidity of 50%, a fixed 12 h light/dark cycle, and *ad libitum* access to food and water. TBI was delivered to mice using a 6-MeV electron linear accelerator (Clinac 2100EX; Varian Medical Systems, Palo Alto, USA) at a fixed dose rate of 2 Gy per minute. Post irradiation, mice were randomized into three groups, where they were administered with an intraperitoneal injection of: 1) 100-μl PBS; 2) 0.1 mM EGFP in 100-μl PBS; or 3) 0.1 mM NeuroD-EGFP in 100-μl PBS. The study was conducted in accordance with protocols approved by the Animal Ethics Committee of Soochow University, China.

### Histology

Smallintestine tissues were isolated at different time points after irradiation, fixed with 10% neutral formalin overnight, and embedded in paraffin. 3 μm-thick paraffin sections were stained with hematoxylin and eosin (H&E). Images of stained tissue sections were captured using an Olympus optical microscope (Olympus, Tokyo, Japan).

Villus height and crypt depth were determined by taking pictures of H&E-stained sections. The images were analyzed using ImageJ software. A minimum of 30 well-oriented, full-length crypt-villus units per mouse were measured. The measurements in pixels were converted to length in μm with the following conversion factor: 1.46 pixels per μm. Crypts per circumference were counted from three separate tubular intestinal H&E-stained slices for each mouse.

### Immunohistochemical (IHC) analysis

Three-μm-thick paraffin sections were deparaffinized and rehydrated, and endogenous peroxidase activity was blocked via incubation with 3% hydrogen peroxide for 15 min at room temperature. Heat-mediated antigen retrieval was performed in sodium citrate buffer (pH 6.0) for 3 min using a pressure cooker. Non-specific-binding sites were blocked via incubation of the sections with 5% bovine serum albumin (BSA) for 30 min at room temperature. Then, the primary antibody (mentioned below) was diluted, whereupon the sections were stained overnight at 4 °C. Next, the sections were stained with a secondary antibody dilution for 1 h at 37 °C. Finally, the chromogenic reaction was developed with DAB and counterstained with hematoxylin.

The primary antibodies used for IHC were a rabbit polyclonal NeuroD antibody (Santa Cruz Biotechnology, Santa Cruz, USA), a Ki67 antibody (Abcam, Cambridge, USA), or a TIMP-1 antibody (Bioss, Beijing, China). The secondary antibody was a ready to use goat-anti rabbit HRP-IgG dilution purchased from Zhongshan Golden Bridge Biotechnology (Beijing, China).

### Terminal deoxynucleotidyl transferase dUTP nick-end labeling (TUNEL) assay

Initially, apoptosis in the intestine tissues of mice was determined using an *in situ* Cell Death Detection Kit (Roche Diagnostic, Mannheim, Germany) completed according to the manufacturer’s instructions. Briefly, paraffin embedded sections were dewaxed and rehydrated, then incubated with TUNEL reaction mixture at 37 °C for 1 h. The nucleus was stained with DAPI, and the sections were analyzed under confocal laser scanning microscope. TUNEL-positive cells displayed brilliant green fluorescence and were quantified in ten fields (400x magnification) per treated mouse.

### Microarray analysis of gene expression

Jejunum tissues were isolated from EGFP- and NeuroD-EGFP treated mice at 12 h after TBI with 9 Gy (n = 3). Total RNA was extracted from jejunum tissues with Trizol reagent (Invitrogen, Carlsbad, USA), and quantified with a NanoDrop Spectrophotometer (Thermo Scientific Inc., Waltham, USA). RNA integrity was assessed by standard denaturing agarose gel electrophoresis.

mRNA expression profiles were microarrayed with Illumina Mouse WG-6 expression beadchips. The microarrays contained more than 45,200-transcript assay probes corresponding to all mouse mRNA sequences currently annotated (Build 36, Release 22). Total RNA was labeled and hybridized under standard conditions according to the manufacturer’s instructions. Genes with a fold change of 2 or greater were subsequently subjected to pathway analysis using Ingenuity Pathway Analysis (Redwood City, USA).

### Quantitative real-time polymerase chain reaction (qRT-PCR)

Total RNA from jejunum tissues was extracted with Trizol (Invitrogen, Carlsbad, USA) and reverse transcribed to cDNA using an oligo (dT)_12_ primer and Superscript II (Invitrogen, Carlsbad, USA). The SYBR green dye (Takara, Otsu, Japan) was used for amplification of cDNA. mRNA levels of Enpp7, Mbl2, Slc13a1 and Slc40a1, as well as that of the internal standard, glyceraldehyde 3-phosphate dehydrogenase (GAPDH), were measured by real-time PCR in triplicate using a Prism 7500 real-time PCR thermocycler (Applied Biosystems, Foster City, USA). Primers specific to the above genes are listed in Supplementary Table S1 online.

### Statistical analysis

Data are presented as means ± SEM. SPSS 17.0 software (SPSS Inc., Chicago, USA) was used to perform statistical analyses. Data with two groups were analyzed using the Student’s *t* test, whereas the data with greater than two groups were assessed by one-way analysis of variance (ANOVA). Comparisons were considered statistically significant if the *P* value was less than 0.05.

## Additional Information

**Accession code**: Microarray data are available on the GEO database: accession number GSE73068.

**How to cite this article**: Li, M. *et al*. Neurogenic differentiation factor NeuroD confers protection against radiation-induced intestinal injury in mice. *Sci. Rep.* **6**, 30180; doi: 10.1038/srep30180 (2016).

## Supplementary Material

Supplementary Information

## Figures and Tables

**Table 1:** Top 10 genes dysregulated within NeuroD-EGFP- and EGFP-treated mice from a 45,200-transcript microarray of small intestinal tissues (NeuroD-EGFP-treated *vs.* EGFP-treated mice).

**Gene name**	**Fold change**	***P*****-value**	**Chromosome**	**Definition**
	**upregulated**			
Enpp7	5.246542	0.026647	11	Ectonucleotide pyrophosphatase/phosphodiesterase 7
Mbl2	3.086312	0.029015	19	Mannose-binding lectin (protein C) 2
Slpi	2.448368	0.000423	2	Secretory leukocyte peptidase inhibitor
Timp1	2.331027	0.021052	X	Tissue inhibitor of metalloproteinase 1
Saa3	2.170989	0.000384	7	Serum amyloid A 3
Lcn2	1.963526	0.001496	2	Lipocalin 2
Slc5a4b	1.855191	0.010979	10	Solute carrier family 5, member 4b
Chi3l1	1.759049	0.002513	1	Chitinase 3-like 1
Wdr92	1.356791	5.99E-05	11	WD repeat domain 92
Sfrp5	1.351067	0.019725	19	Secreted frizzled-related sequence protein 5
	**downregulated**			
Slc13a1	−4.00218	0.001986	6	Solute carrier family 13, member 1
Slc40a1	−2.91334	0.009675	1	Solute carrier family 40, member 1
Rsad2	−2.41762	0.019864	12	Radical S-adenosyl methionine domain containing 2
AU018778	−2.01096	0.008233	8	Expressed sequence AU018778
D11Lgp2e	−2.00538	0.043613	11	DEXH (Asp-Glu-X-His) box polypeptide 58
Gsta2	−2.00401	0.004064	9	Glutathione S-transferase, alpha 2
Amy2-2	−2.00252	0.037473	3	Amylase 2-2, pancreatic
Gsta1	−2.0009	0.004043	9	Glutathione S-transferase, alpha 1
EG432555	−1.97716	0.011818	11	Predicted gene, EG432555
Slc16a9	−1.94278	0.041652	10	Solute carrier family 16, member 9

## References

[b1] Liauw S. L., Connell P. P. & Weichselbaum R. R. New paradigms and future challenges in radiation oncology: an update of biological targets and technology. Sci. Transl. Med. 5, 173sr172 (2013). 10.1126/scitranslmed.3005148 PMC3769139 23427246

[b2] Christensen D. M., Iddins C. J., Parrillo S. J., Glassman E. S. & Goans R. E. Management of ionizing radiation injuries and illnesses, part 4: acute radiation syndrome. J. Am. Osteopath. Assoc. 114, 702–711 (2014). 25170040 10.7556/jaoa.2014.138

[b3] Prasanna P. G. . Radioprotectors and Radiomitigators for Improving Radiation Therapy: The Small Business Innovation Research (SBIR) Gateway for Accelerating Clinical Translation. Radiat. Res. 184, 235–248 (2015). 26284423 10.1667/RR14186.1 PMC4581592

[b4] Williams J. P. . Animal models for medical countermeasures to radiation exposure. Radiat. Res. 173, 557–578 (2010). 20334528 10.1667/RR1880.1 PMC3021126

[b5] Potten C. S. A comprehensive study of the radiobiological response of the murine (BDF1) small intestine. Int. J. Radiat. Biol. 58, 925–973 (1990). 1978853 10.1080/09553009014552281

[b6] Potten C. S. Radiation, the ideal cytotoxic agent for studying the cell biology of tissues such as the small intestine. Radiat. Res. 161, 123–136 (2004). 14731078 10.1667/rr3104

[b7] Yeoh E. . Effect of pelvic irradiation on gastrointestinal function: a prospective longitudinal study. Am. J. Med. 95, 397–406 (1993). 8213872 10.1016/0002-9343(93)90309-d

[b8] Gasa R. Transcriptional control of pancreatic endocrine cell development. Drug News Perspect. 18, 567–576 (2005). 16421630 10.1358/dnp.2005.18.9.953669

[b9] Cho J. H. & Tsai M. J. The role of BETA2/NeuroD1 in the development of the nervous system. Mol. Neurobiol. 30, 35–47 (2004). 15247487 10.1385/MN:30:1:035

[b10] Naya F. J., Stellrecht C. M. & Tsai M. J. Tissue-specific regulation of the insulin gene by a novel basic helix-loop-helix transcription factor. Genes Dev. 9, 1009–1019 (1995). 7774807 10.1101/gad.9.8.1009

[b11] Lee J. E. . Conversion of Xenopus ectoderm into neurons by NeuroD, a basic helix-loop-helix protein. Science 268, 836–844 (1995). 7754368 10.1126/science.7754368

[b12] Lee J. E. Basic helix-loop-helix genes in neural development. Curr. Opin. Neurobiol. 7, 13–20 (1997). 9039799 10.1016/s0959-4388(97)80115-8

[b13] Farah M. H. . Generation of neurons by transient expression of neural bHLH proteins in mammalian cells. Development 127, 693–702 (2000). 10648228 10.1242/dev.127.4.693

[b14] Naya F. J. . Diabetes, defective pancreatic morphogenesis, and abnormal enteroendocrine differentiation in BETA2/neuroD-deficient mice. Genes Dev. 11, 2323–2334 (1997). 9308961 10.1101/gad.11.18.2323 PMC316513

[b15] Miyata T., Maeda T. & Lee J. E. NeuroD is required for differentiation of the granule cells in the cerebellum and hippocampus. Genes Dev. 13, 1647–1652 (1999). 10398678 10.1101/gad.13.13.1647 PMC316850

[b16] Chen J. . A novel type of PTD, common helix-loop-helix motif, could efficiently mediate protein transduction into mammalian cells. Biochem. Biophys. Res. Commun. 347, 931–940 (2006). 16870135 10.1016/j.bbrc.2006.06.173

[b17] Noguchi H., Bonner-Weir S., Wei F. Y., Matsushita M. & Matsumoto S. BETA2/NeuroD protein can be transduced into cells due to an arginine- and lysine-rich sequence. Diabetes 54, 2859–2866 (2005). 16186386 10.2337/diabetes.54.10.2859

[b18] Huang Y. . Reversal of hyperglycemia by protein transduction of NeuroD *in vivo*. Acta Pharmacol. Sin. 28, 1181–1188 (2007). 17640481 10.1111/j.1745-7254.2007.00626.x

[b19] Waselenko J. K. . Medical management of the acute radiation syndrome: recommendations of the Strategic National Stockpile Radiation Working Group. Ann. Intern. Med. 140, 1037–1051 (2004). 15197022 10.7326/0003-4819-140-12-200406150-00015

[b20] Berger M. E., Christensen D. M., Lowry P. C., Jones O. W. & Wiley A. L. Medical management of radiation injuries: current approaches. Occup. Med. (Lond) 56, 162–172 (2006). 16641501 10.1093/occmed/kql011

[b21] Gosselin T. K. & Mautner B. Amifostine as a radioprotectant. Clin. J. Oncol. Nurs. 6, 175–176, 180 (2002). 11998614 10.1188/02.CJON.175-176

[b22] Arpino V., Brock M. & Gill S. E. The role of TIMPs in regulation of extracellular matrix proteolysis. Matrix Biol. 44–46, 247–254 (2015). 10.1016/j.matbio.2015.03.005 25805621

[b23] Bertaux B., Hornebeck W., Eisen A. Z. & Dubertret L. Growth stimulation of human keratinocytes by tissue inhibitor of metalloproteinases. J. Invest. Dermatol. 97, 679–685 (1991). 1940438 10.1111/1523-1747.ep12483956

[b24] Hayakawa T., Yamashita K., Tanzawa K., Uchijima E. & Iwata K. Growth-promoting activity of tissue inhibitor of metalloproteinases-1 (TIMP-1) for a wide range of cells. A possible new growth factor in serum. FEBS Lett. 298, 29–32 (1992). 1544418 10.1016/0014-5793(92)80015-9

[b25] Saika S. . Recombinant TIMP-1 and -2 enhance the proliferation of rabbit corneal epithelial cells *in vitro* and the spreading of rabbit corneal epithelium *in situ*. Curr. Eye Res. 17, 47–52 (1998). 9472470 10.1076/ceyr.17.1.47.5247

[b26] Wang T., Yamashita K., Iwata K. & Hayakawa T. Both tissue inhibitors of metalloproteinases-1 (TIMP-1) and TIMP-2 activate Ras but through different pathways. Biochem. Biophys. Res. Commun. 296, 201–205 (2002). 12147251 10.1016/s0006-291x(02)00741-6

[b27] Rossi L. . The tissue inhibitor of metalloproteinases 1 increases the clonogenic efficiency of human hematopoietic progenitor cells through CD63/PI3K/Akt signaling. Exp. Hematol. 43, 974–985 (2015). 26213230 10.1016/j.exphem.2015.07.003

[b28] Lu Y. . Tissue inhibitor of metalloproteinase-1 promotes NIH3T3 fibroblast proliferation by activating p-Akt and cell cycle progression. Mol. Cells 31, 225–230 (2011). 21350939 10.1007/s10059-011-0023-9 PMC3932703

[b29] Guedez L. . *In vitro* suppression of programmed cell death of B cells by tissue inhibitor of metalloproteinases-1. J. Clin. Invest. 102, 2002–2010 (1998). 9835626 10.1172/JCI2881 PMC509153

[b30] Han X., Sun Y., Scott S. & Bleich D. Tissue inhibitor of metalloproteinase-1 prevents cytokine-mediated dysfunction and cytotoxicity in pancreatic islets and beta-cells. Diabetes 50, 1047–1055 (2001). 11334407 10.2337/diabetes.50.5.1047

[b31] Vorotnikova E., Tries M. & Braunhut S. Retinoids and TIMP1 prevent radiation-induced apoptosis of capillary endothelial cells. Radiat. Res. 161, 174–184 (2004). 14731072 10.1667/rr3107

[b32] Chromek M., Tullus K., Lundahl J. & Brauner A. Tissue inhibitor of metalloproteinase 1 activates normal human granulocytes, protects them from apoptosis, and blocks their transmigration during inflammation. Infect. Immun. 72, 82–88 (2004). 14688084 10.1128/IAI.72.1.82-88.2004 PMC343974

[b33] Liu X. W., Bernardo M. M., Fridman R. & Kim H. R. Tissue inhibitor of metalloproteinase-1 protects human breast epithelial cells against intrinsic apoptotic cell death via the focal adhesion kinase/phosphatidylinositol 3-kinase and MAPK signaling pathway. J. Biol. Chem. 278, 40364–40372 (2003). 12904305 10.1074/jbc.M302999200

[b34] Liu X. W. . Tissue inhibitor of metalloproteinase-1 protects human breast epithelial cells from extrinsic cell death: a potential oncogenic activity of tissue inhibitor of metalloproteinase-1. Cancer Res. 65, 898–906 (2005). 15705888

[b35] Guo L. J. . Tissue inhibitor of matrix metalloproteinase-1 suppresses apoptosis of mouse bone marrow stromal cell line MBA-1. Calcif. Tissue Int. 78, 285–292 (2006). 16691494 10.1007/s00223-005-0092-x

[b36] Mutoh H. . The basic helix-loop-helix transcription factor BETA2/NeuroD is expressed in mammalian enteroendocrine cells and activates secretin gene expression. Proc. Natl. Acad. Sci. USA 94, 3560–3564 (1997). 9108015 10.1073/pnas.94.8.3560 PMC20478

[b37] Poulin G., Turgeon B. & Drouin J. NeuroD1/beta2 contributes to cell-specific transcription of the proopiomelanocortin gene. Mol. Cell. Biol. 17, 6673–6682 (1997). 9343431 10.1128/mcb.17.11.6673 PMC232521

[b38] Harfouche G. & Martin M. T. Response of normal stem cells to ionizing radiation: a balance between homeostasis and genomic stability. Mutat. Res. 704, 167–174 (2010). 20117235 10.1016/j.mrrev.2010.01.007

[b39] Noguchi H. . Induction of pancreatic stem/progenitor cells into insulin-producing cells by adenoviral-mediated gene transfer technology. Cell transplant. 15, 929–938 (2006). 17299998 10.3727/000000006783981431

[b40] Guo Q. S. . Combined transfection of the three transcriptional factors, PDX-1, NeuroD1, and MafA, causes differentiation of bone marrow mesenchymal stem cells into insulin-producing cells. Exp. Diabetes Res. 2012, 672013 (2012). 22761608 10.1155/2012/672013 PMC3385644

[b41] Schonhoff S. E., Giel-Moloney M. & Leiter A. B. Minireview: Development and differentiation of gut endocrine cells. Endocrinology 145, 2639–2644 (2004). 15044355 10.1210/en.2004-0051

