# Supplementary material for: Neurogenic differentiation factor NeuroD confers protection against radiation-induced intestinal injury in mice
Source: Sci Rep. 2016 Jul 20;6:30180. doi: 10.1038/srep30180 (PMC4951798; doi:10.1038/srep30180)
Supplement: Supplementary Information [file srep30180-s1.pdf]

Supplementary information

Title: Neurogenic differentiation factor NeuroD confers protection against radiation-induced intestinal injury in mice

By: Ming Li, Aonan Du, Jing Xu, Yanchao Ma, Han Cao, Chao Yang, Xiao-Dong Yang, Chun-Gen Xing, Ming Chen, Wei Zhu, Shuyu Zhang, Jianping Cao

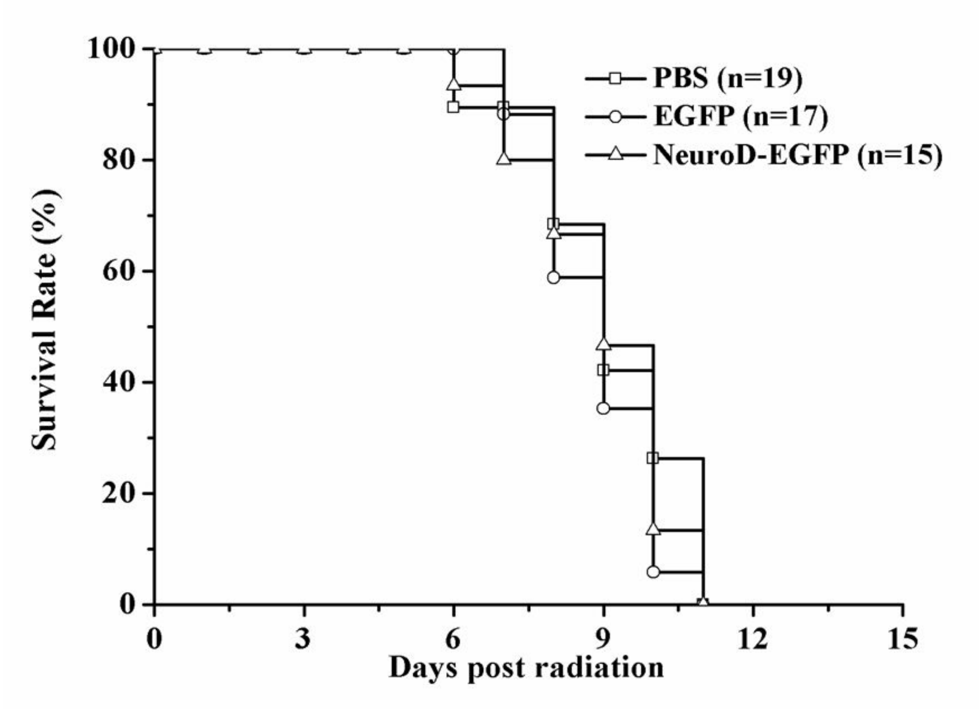

Supplementary Figure S1

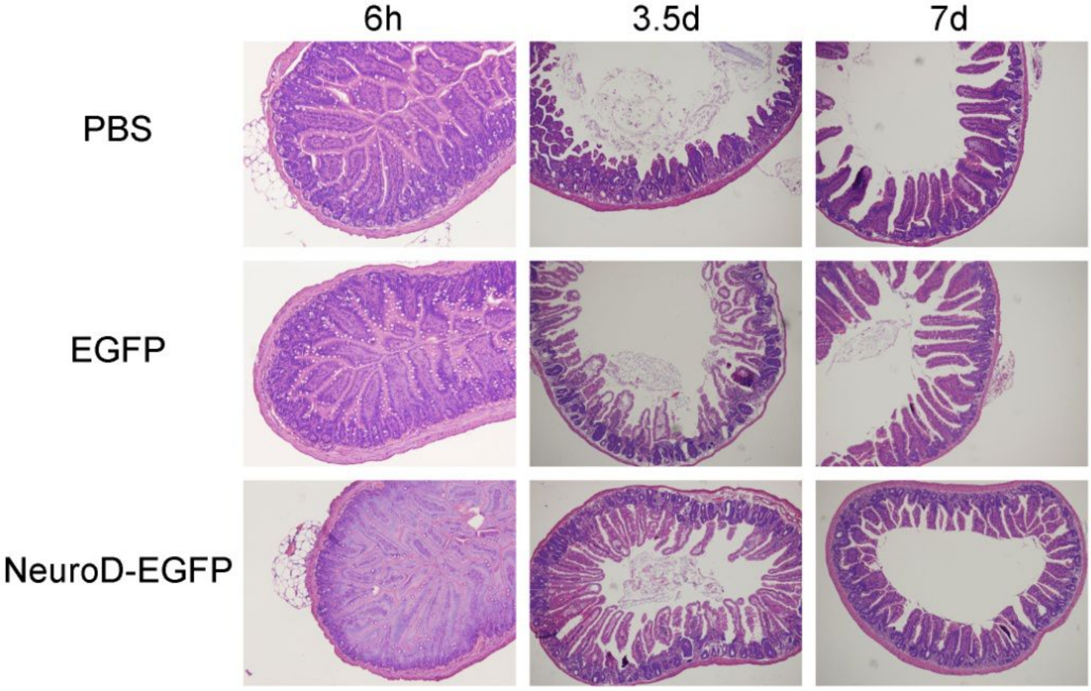

Supplementary Figure S2

Supplementary Table S1 Primer sequences for real-time PCR analysis.

**Gene**	**Forward primer**	**Reverse primer**
Enpp7	5’- CAGGATCACACCTCTGCTCA-3’	5’- ACTCTCGAAGGGTTCCACCT-3’
Mbl2	5’- GGTTAAAAGGAGCAGTGGGA-3’	5’- CTCAGCTCTGATCGTAGGGC-3’
Slc40a1	5’- CTGGATTGTTGTTGTGGCAG-3’	5’- CCAGGATGTTGGTTAGCTGG-3’
Slc13a1	5’-TGCATTTCTTCTGCCAGTTG-3’	5’-TGTTTGTCATGGCATGGTCT-3’
GAPDH	5’-CGTCCCGTAGACAAAATGGT-3’	5’-TTGATGGCAACAATCTCCAC-3’

Figure legends

Supplementary Figure S1 Kaplan-Meier survival analyses of C57BL/6J mice intraperitoneally injected with PBS, EGFP or NeuroD-EGFP following 9 Gy TBI.

Supplementary Figure S2 Representative H&E staining of intestinal sections from PBS, EGFP or NeuroD-EGFP treated mice at 6 h, 3.5 and 7 days after 9 Gy TBI (100× or 200× magnification).

Supplementary Table S1 Primer sequences for real-time PCR analysis.
